# In vivo observation of gold nanoparticles in the central nervous system of *Blaberus discoidalis*

**DOI:** 10.1186/1477-3155-9-5

**Published:** 2011-02-18

**Authors:** Aracely Rocha, Yan Zhou, Subrata Kundu, Jorge M González, S BradleighVinson, Hong Liang

**Affiliations:** 1 Department of Mechanical Engineering, Texas A&M University, College Station, Texas, USA; 2 Materials Science and Engineering, Texas A&M University, College Station, Texas, USA; 3 Department of Entomology, Texas A&M University, College Station, Texas, USA

## Abstract

**Background:**

Nanoparticles (NPs) are widely studied for biomedical applications. Understanding interactions between NPs and biomolecules or cells has yet to be achieved. Here we present a novel *in vivo *method to study interactions between NPs and the nervous system of the discoid or false dead-head roach, *Blaberus discoidalis*. The aims of this study were to present a new and effective method to observe NPs *in vivo *that opens the door to new methods of study to observe the interactions between NPs and biological systems and to present an inexpensive and easy-to-handle biological system.

**Results:**

Negatively charged gold nanoparticles (nAuNPs) of 50 nm in diameter were injected into the central nervous system (CNS) of the insect. By using such a cost effective method, we were able to characterize nAuNPs and to analyze their interactions with a biological system. It showed that the charged particles affected the insect's locomotion. The nAuNPs affected the insect's behavior but had no major impacts on the life expectancy of the cockroach after two months of observation. This was apparently due to the encapsulation of nAuNPs inside the insect's brain. Based on cockroach's daily activity, we believed that the encapsulation occurred in the first 17 days.

**Conclusions:**

The method proposed here is an inexpensive and reliable way of observing the response of biological systems to nanoparticles in-vivo. It opens new windows to further understand how nanoparticles affect neural communication by monitoring insect activity and locomotion.

## Background

Due to their small size, nanoparticles (NPs) have been used to probe biological systems [1-3]. Common biological systems, mainly mice, currently used to study, analyze, and test *in vivo *treatments for neuron damage and repair are expensive and many times difficult to maintain. It is necessary to find a suitable biological system that is inexpensive, easy to maintain, and handle. As early as in 1990, Huber et al. reported cockroaches as good candidates for neurobiology studies [4]. This idea was later applied by Scharrer for endocrine studies [5]. There are reports proving the similarities between vertebrate and invertebrate brains [6]. In particular, non-vertebrate systems such as cockroaches were ideal models for neurotoxicology studies [7]. The comparison between invertebrate (like cockroaches) and vertebrate (like mice) has been made in terms of their behavior, anatomy, biology, and physiology. Invertebrate subjects are not only cost effective and readily available, but also they do not feel pain [8]. This opens new avenues for experimental protocols and controls currently implemented in vertebrate animals and humans.

Cockroaches have been used as model systems for neurological research. Early neurobiology cockroach research has been focused on octopamine and serotonin response in the nervous system (NS). Previous studies were to observe how chemicals were distributed in the brain and how they affected the nervous system [9,10]. In more recent work by Brown et al., roaches have been used to study the effects of age on memory and brain integrity [11].

The use of nanoparticles in biological systems is a subject that has been under scrutiny for some time. The use of nanoparticles for imaging and drug delivery has been extensively studied in mice. Hainfeld and colleagues have used gold nanoparticles to enhance radiotherapy in mice and as a contrast agent for X-ray imaging [12,13]. Functionalized gold nanoparticles have also been used to investigate targeted drug delivery [14-16]. However, these *in vivo *methods have not been applied for simpler and inexpensive biological systems like insects.

In the present work, we use *Blaberus discoidalis*, a neotropical cockroach, as the model system. We study the effects and interactions of negatively charged gold nanoparticles (nAuNPs) with the cockroaches CNS *in vivo*. The authors refer to the nervous system as the brain and the nerve cord as described in the American Cockroach by Bell [17]. Negatively charged nanoparticles were selected to enhance nanoparticle interaction with the nervous system during signal transfer i.e. during a nerve impulse.

## Methods

A new method to introduce nanoparticles into the nervous system (NS) of *Blaberus discoidalis *roaches was used. This method allowed us to study effects of nanoparticles on the roach's CNS *in vivo*. Two groups of roaches were selected for this study. Each group had 9 individuals. The selected groups were separated for 24 hours prior to the treatment. Group 1 served as control; no nanoparticles were injected into this group. Group 2 was treated with negatively charged spherical gold nanoparticles (nAuNPs) of 50 nm in diameter.

Male *Blaberus discoidalis *(weight = 2.1 ± 0.3 g) grown in-house were used in this study. These roaches were maintained in hard plastic containers (9 × 18") inside an environment controlled room with a temperature of 28 ± 2°C and a 12/12 h day/night cycle. They were fed with Dry dog chow. Food and water were supplied *ad libitum*.

The cockroaches were kept in isolation to minimize stressors like noise, wind, and vibration that could alter their behavior. A two-minute video was taken daily at 8:00 am, only 10 minutes into the light cycle, to record their activity. Although the insect is most active during the dark cycle, light was needed to record their activity. The first hour was selected for recording since slightly over one third or 38.1% of the cockroaches show activity during the first hour of the light cycle [17,18].

**Figure 1:**
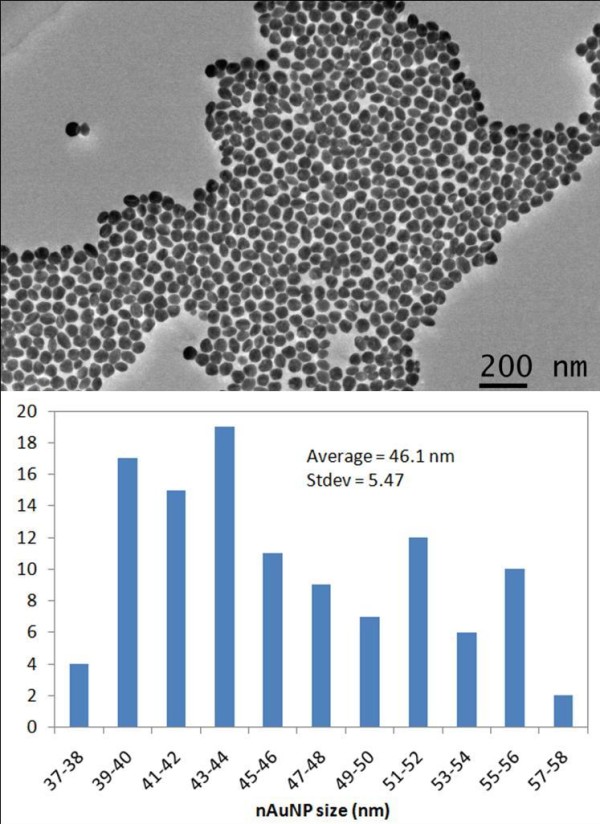
Negatively charged gold nanoparticles (nAuNP) size distribution & analysis.

The nanoparticles were ~50 nm in diameter. They were synthesized using the well known Turkevich method [19]. The synthesized Au particles were stabilized and separated from each other by the negatively charged tri-sodium-citrate molecule. Their size was controlled by the reaction time and the amount of gold atoms present in the solution. This method delivers 95% of spherical particles and no further treatment was done to eliminate the remaining 5% of non-spherical nanoparticles. The average particle size is 46.7 nm ± 5.47 nm as verified by JEOL-JEM 2010 TEM and analyzed with Image J. The particle size distribution image and analysis is summarized in Figure 1. The particles were suspended in DI water with a concentration of 1 × 10^11 ^nanoparticles/mL. They were then coated with tri-sodium citrate molecules to create a negatively charged surface. The charge was to avoid agglomeration, ensure suspension in the solution, and to promote their interactions with the CNS.

According to Patil and colleagues [20] and Tim and colleagues [21], the zeta potential values for gold nanoparticles prepared by this method are stable and strongly dependent on nanoparticle size. The zeta potential for a 47.1 nm gold nanoparticle prepared by this method is -32.65 mV [21]. The negatively charged gold nanoparticles are also fluorescent. The 50 nm particles used absorb a light wave of 510 nm and emit at 560 nm [22-24]. This allows for fluorescent and spectral imaging to identify the presence of nAuNPs in the tissue without adding fluorescent tags.

### Nanoparticle introduction to the CNS

**Figure 2:**
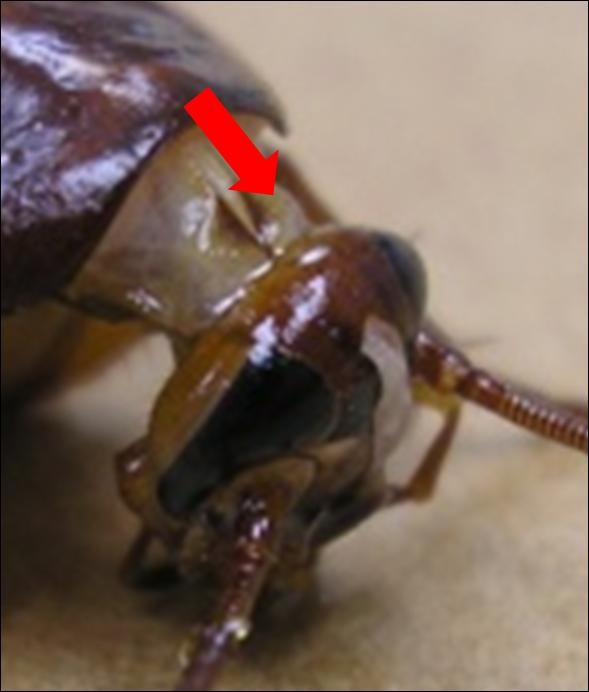
Nanoparticle injection site and direction is indicated with the red arrow.

The nAuNPs were introduced in the CNS through an injection between the brain and the sub esophageal ganglion (SEG) through the neck in the direction shown in Figure 2. A 1 cc syringe with 30 gauge needle was used to inject the nAuNPs suspended in DI water. The cockroach was immobilized by exposing it to a CO_2 _atmosphere until no signs of motion were observed (approximately 30 s). The needle was inserted 1.5 to 2 mm into the neck in the location and direction shown in Figure 2, allowing to reach the brain of the insect. A stepper motor with speed and time control was used to inject 7 μL of nAuNPs/DI water solution, giving 7 × 10^11 ^nanoparticles injected into each cockroach.

The roaches were placed in the plastic container immediately after treatment and were closely monitored for the first 4 hours to ensure activity had been resumed. The insects were monitored daily to verify activity. The roaches that did not show signs of activity were considered dead and were removed and placed in a -80°C freezer to prevent tissue damage and allow further analysis. After two months, 7 cockroaches from the control and 6 cockroaches from the treated group were alive, giving 78% and 67% survival rates respectively. The activity recording was stopped at two months and two cockroaches from the nAuNPs treated group and two from the control group were sacrificed and their brains dissected for analysis. The remaining cockroaches from each group were sacrificed by freezing at -80°C.

#### Imaging and testing

Four instruments were used to analyze the presence of nAuNPs in the cockroach's brain and to study the interactions between nAuNPs and the brain tissue: hyperspectral imaging, XPS, confocal microscopy, and TEM imaging. The Hyperspectral imaging from CytoViva was used to identify the organs affected by the nAuNPs. The XPS was used to verify the presence of nAuNPs embedded in the brain tissue. The confocal microscope and TEM were used to gain insights into the interaction of nAuNPs and the insect's CNS.

#### Sample preparation

Sample preparation varied with each test system. The two nAuNPs treated cockroaches prepared for Hyperspectral imaging were dissected to remove the organs in the thorax and head. The organs removed included the brain, antennae, fat bodies, esophagus, malphigian tubules, and haemolymph. The organs were fixed with Zamboni's fixative (Newcomer Supply) for 10 minutes and rinsed with DPBS 3 times for 5 minutes. The samples were allowed to air dry over a 25 mm glass cover slip.

The samples prepared for XPS, Confocal microscopy, and TEM imaging were obtained from frozen sections. The cockroach's head was removed and the brain extracted. The brain was rinsed with DPBS and fixed with FrozFix (Newcommer Supply) for two hours to allow thorough diffusion of the fixative in the brain tissue. The brain was then mounted in Optical coherence tomography (OCT, Fischer Scientific) and allowed to harden at -17°C. The samples were sliced to 12 μm thickness with a cryocutter. The slices were collected on 1in^2 ^quartz microscope slides for XPS analysis. The samples prepared for confocal microscopy were mounted on positively charged microscope slides under DPBS media and covered with a glass cover slip. The samples for TEM imaging were placed on copper grids and allowed to dry for imaging.

## Results

### Cockroach activity

**Figure 3:**
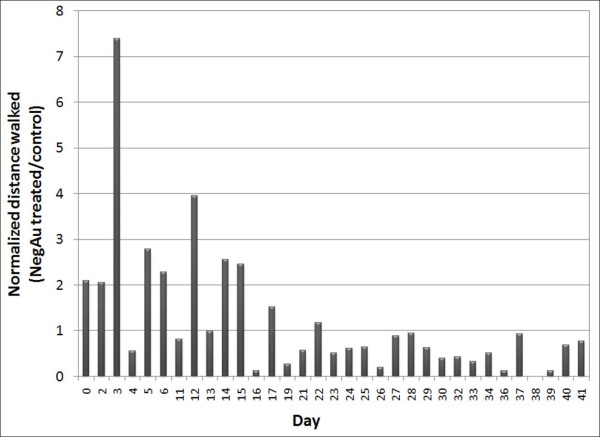
Normalized (nAuNPs treated/untreated) activity.

The cockroach activity was recorded by measuring the total distance walked by each group daily. Two-minute video recordings were performed at the beginning of the light cycle at 8:00 am for six weeks. This time is chosen because it is when the insects are most active under light. The motion of each cockroach was traced with Image Tool and the distance walked was calculated by comparing with a fixed reference of known size in the container. The results of cockroach activity are summarized in Figure 3. The days not shown in the summary are due to video recording device failure or due to corrupt video files.

There are several possible factors affecting insects' activity. Reproductive cycle, age, temperature, humidity, wind, noise, vibration, and changes in weather are just a few examples [17]. The variation due to the reproductive cycle and age was eliminated by using only young males in this study. The effects of temperature, humidity, and wind were diminished by maintaining them in a controlled environment. However, the fluctuations in noise, vibration and changes in weather affect the activity of both groups. The effects of these variables are diminished by presenting the activity ratio of the treated to the untreated group. Although the treated/untreated ratio still shows variations (days 4, 11, and 13 in particular), Figure 3 indicates an increased activity for the nAuNPs treated group for 17 days following treatment. After 17 days, their activity falls below that of the control group. After two months, 7 cockroaches from the control and 6 cockroaches from the treated group were alive, giving 78% and 67% survival rate respectively. The observation period was terminated at 2 months since there were no visible differences in the cockroaches' behavior after day 17.

What is the reason behind this? To understand the effects of nAuNPs on the insects' behavior, we conducted a series of characterization experiments for NPs with surrounding tissues. Spectroscopic and morphologic analyses were conducted using hyperspectral imaging, XPS, Confocal microscopy, and TEM. Using these tools we identified the location and interactions of the nAuNPs with the cockroach's CNS.

### Spectroscopic analysis

**Figure 4:**
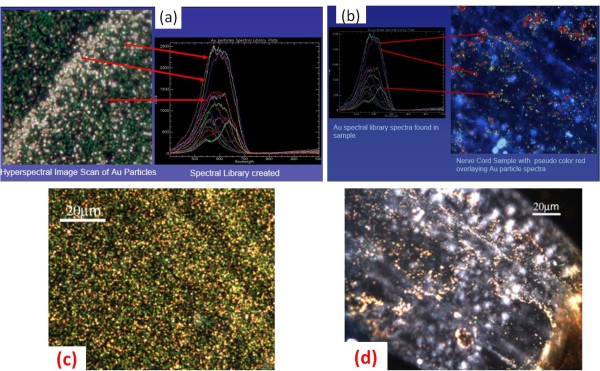
**Hyperspectral imaging of NP solution and treated nervous system**. (a) Negative gold nanoparticle hyperspectral imaging. (b) Spectral scan of brain and nerve cord. (c) Scan areas for nAuNPs/DI water solution spectra. (d) Scan area of treated nerve cord

The hyperspectral imaging system from CitoViva was used to identify the location of the nAuNPs particles in the tested roach. This imaging system identified the presence of gold in the tissues by comparison. A sample of nAuNPs/DI water solution was scanned to identify the emitted fluorescence of the nanoparticles. The hyperspectral imaging, as shown in Figure 4a, provided a range of emitted signal due to the variations in size during nanoparticle fabrication and possible agglomeration once in contact with the CNS. A signal library was generated from this scan, Figure 4a. The nAuNPs treated tissue was then scanned and the spectral imaging was compared to that of the library. From the scanned tissues, only the spectra recorded from the brain and nerve cord matched to that of the library generated from the nAuNPs/DI water solution. Results are shown in the Figure 4b. The optical images of the scanned regions are shown in the Figures 4c and 4d and correspond to Figures 4a and 4b respectively.

**Figure 5:**
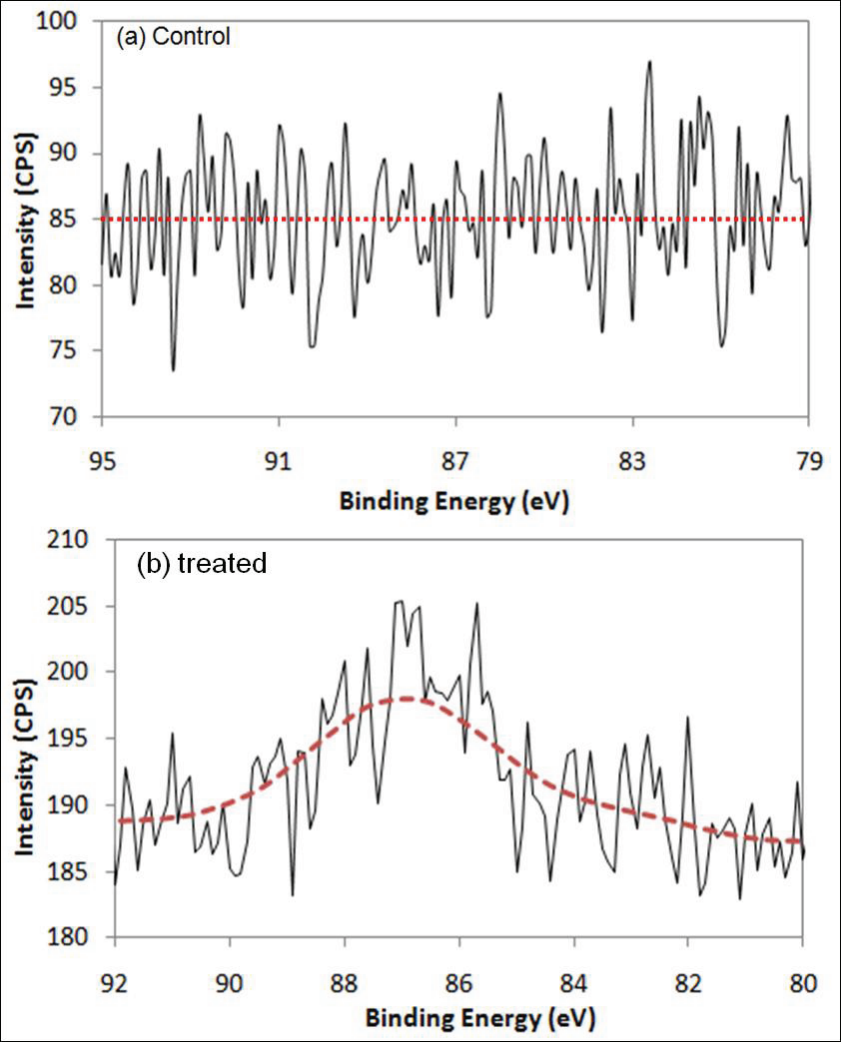
**Gold has a bonding energy of 85 eV**. (a) XPS results for control cockroach brain. (b) XPS results for nAuNPs treated cockroach brain.

A Kratos Axis Ultra Imaging X-ray photoelectron spectrometer (XPS) with a spherical mirror analyzer was used in this study. It was operated with a Mg-Kα (1253.6 eV) X-ray radiation at a power of 350 W and a base pressure of 10^-10 ^Torr. The XPS system was used to verify the presence of the nanoparticles inside the brain by scanning the cryocut and fixed cockroach brain slices mounted on quartz slides. A control and a nAuNPs treated brains were scanned for comparison. Figure 5a shows the results for the control sample and Figure 5b for the nAuNPs treated brain. The binding energy for gold is at 85 eV.

The high signal-to-noise ratio of the XPS scans was caused by too few particles on the scanned surface. The samples used for these scans were 12 μm thick slices that were cryocut from the cockroach brain. The XPS could only scan to a few nanometers (<10 nm) deep from the surface. This limited the number of nAuNPs present in the scanned region since only a few nanoparticles were exposed within 10 nm from the surface. Interestingly, the difference between the control and the nAuNPs treated samples were seen around 85 eV. The curve fitting obtained for Figure 5b was obtained by averaging of 21 consecutive intensity readings (10 above and 10 below) for each binding energy value recorded. This allows for a moving average and smoothing of the fitted curve. The XPS results indicated that the gold nanoparticles were dispersed inside the insect's brain.

### Morphological analysis

#### Microscopic imaging

**Figure 6:**
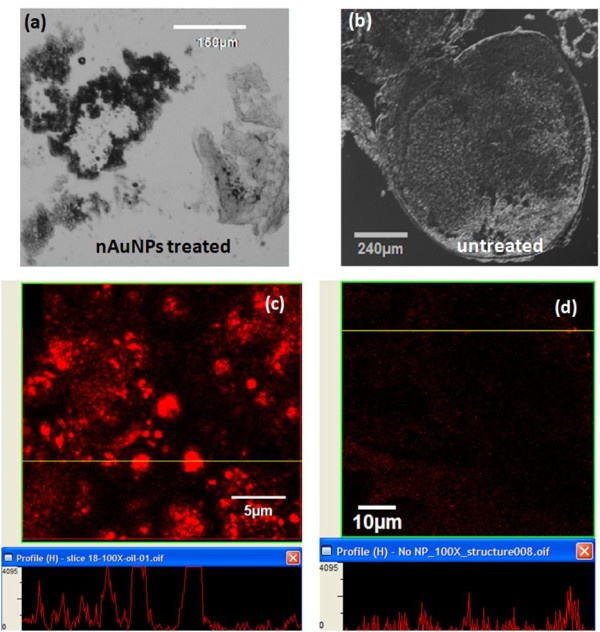
**TEM of treated and untreated brains**. Transmission light image of (a) nAuNPs treated dissected cockroach brain and (b) control. Darker tissue is a sign of nanoparticles. A clear difference can be observed in the treated tissue (a) while the untreated (b) shows no difference in the tissue. Fluorescent image of (c) nAuNPs trated and (d) untreated samples. The lower window shows the fluorescent intensity at the location of the yellow line on the upper windows.

An Olympus FV1000 Confocal Microscope equipped with a 510 nm argon laser was used to detect where the nAuNPs were located within the brain. The samples were fixed and cryocut to 12 μm thickness and mounted with DPBS (Dulbecco's Phosphate Buffered Saline). The gold nanoparticles used in this study fluoresced at 560 nm with an excitation wavelength of 510 nm. In the transmission images, Figure 6a and 6b, exhibited visible differences in the tissues of the nAuNPs treated and untreated brains respectively. The darker regions were an indication of nanoparticle dispersion within the tissue.

The electron transmission microscopic image showed a clear difference between the treated and untreated cockroach brains. The nAuNPs treated brain had an abnormal tissue (dark) due to the embedded nanoparticles. This further proved the existence nAuNPs inside the cockroach's brain. Figure 6c and 6d show the fluorescence of the treated and untreated brains respectively. The main challenge of the fluorescent images was the self fluorescence of the cockroach brain tissues. The self fluorescence was absorbed and emitted at a wavelength close to that of the gold nanoparticles. However, it was clear that the nAuNPs treated brain had stronger fluorescence intensity than the control. The horizontal yellow line on the top images of Figures 6c and 6d showed the location of the intensity profile below. These locations were selected because they exhibit the highest intensity. The fluorescence of the treated brain was significantly higher than that of the untreated brain. The intensity difference was further enhanced by the fact that the laser power was set at 30% for the treated brain and 50% for the untreated brain, i.e. the fluorescent signal recorded for the untreated brain was partially due to the higher laser power and the self fluorescence of the tissue.

#### Nanoscopic imaging

**Figure 7:**
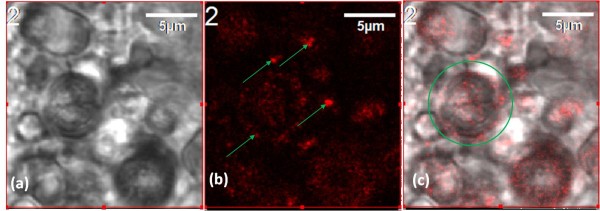
**(a) Transmission, (b) fluorescent, and (c) overly image of nAuNPs treated brain**. Particle encapsulation is evident. The arrows in (b) indicate particle agglomerations.

**Figure 8:**
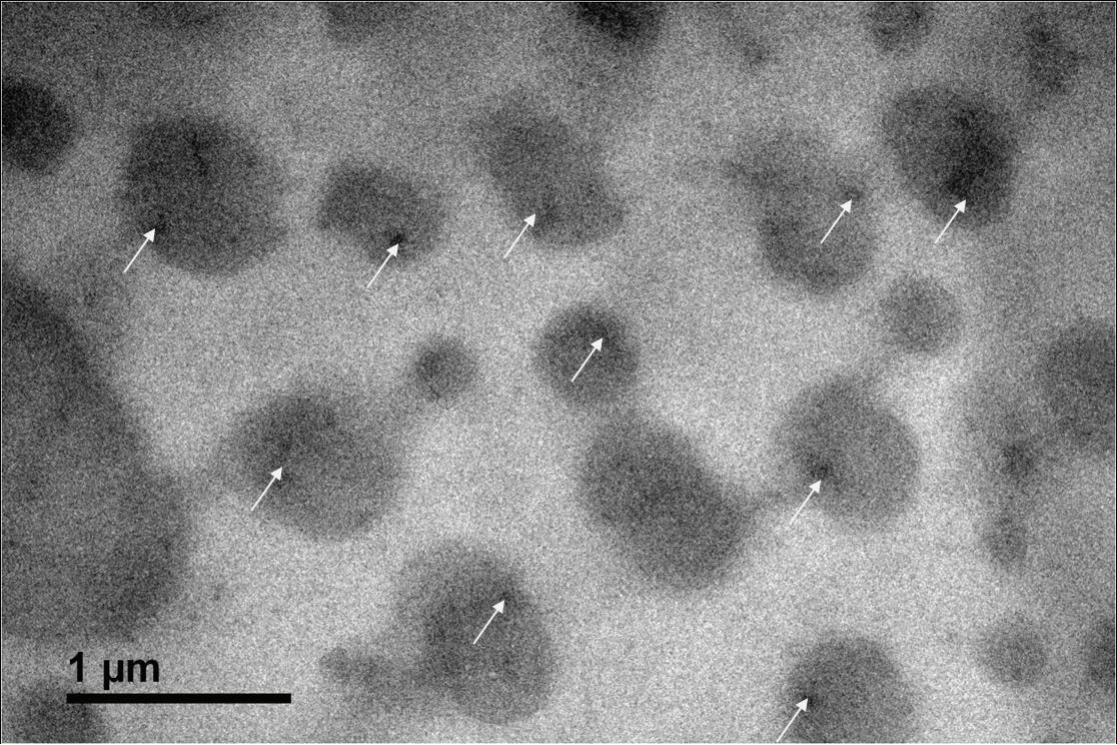
**TEM image of nAuNPs treated brain confirms nanoparticle encapsulation by the brain tissue**. The arrows indicate the nanoparticle inside the protein capsule.

Upon closer inspection of the treated brain tissue, there was evidence of nanoparticle encapsulation. Figure 7a showed well-defined 2-5 μm (2000 to 5000 nm) diameter spheres. Upon inspection of the fluorescent image of this view, Figure 7b, hundreds of small nanoparticles were found dispersed or agglomerated (indicated with green arrows) inside these spheres. Figure 7c, an overlay of the transmission (6a) and fluorescent (6b) images further proved the agglomeration of nanoparticles inside the spherical structures. A JEOL-JEM 2010 TEM was used to characterize the morphology of NPs in the cockroaches' brain. Figure 8 showed nAuNPs (in dark) surrounded by light colored spheres, i.e., the nanoparticles were encapsulated. The spheres in Figure 8 ranged from 200 to 500 nm in diameter. This value disagreed with by one order of magnitude to that observed in Figure 7. In Figure 8, we observed a single nanoparticle embedded in a sphere of 200-500 nm in diameter while Figure 7 shows an agglomeration of these smaller spheres into larger ones of approximately 2-5 μm in diameter. This indicates a multi-level self-arrangement of embedded nanoparticles. Based on studies by Cedervall et al. [25] and Lundqvist and colleagues [26], it is known that the nanoparticles will interact with the proteins present in the biological system, i.e. the material surrounding the nanoparticles are proteins present in the nervous system of the cockroach.

## Discussion

The results of characterization have repeatedly proven that the nAuNPs were encapsulated. How did this process occur? There are two possible reasons [1], a defense mechanism of the immune system of the cockroach against a foreign object, or [2] as a protein corona that surrounds the nanoparticles due to its negative surface charge. In terms of defense mechanisms, when a foreign object enters the biological system, the response of the immune system is to block further damage by encapsulating the object. This response has been readily found and studied in insects [27,28]. The immune system surrounds the foreign object by phagocytes to then be digested and/or destroyed. Some parasites avoid encapsulation due to an ionic surface. When these parasites were rinsed to remove the ions from the surface, encapsulation happened [29]. Once encapsulated, the foreign objects were expected to either reduce in size or change morphologically. In the present research, the nanoparticles are small enough (50 nm) to be encapsulated by phagocytosis. Through this process the immune system will excrete the nanoparticle from the cell. It is evidenced in Figure 6b that the nAuNPs nanoparticles remain inside the cells after 2 months of injection. In the present research, we only observed nanoparticle encapsulation with no visible changes in particle size or morphology, as shown in Figure 8. It is seen that particles are well defined spheres of approximately 50 nm diameter. It has been reported that a protein corona is the encapsulation of charged particles by the polar amino acids in proteins [25,27,30]. When the charged nanoparticles come in contact with live tissue, the proteins or amino acids of opposite charge will be attracted to the surface of the particle. This immediate attraction might affect the normal behavior of other proteins whose function or processes depended on the protein now in contact with the nanoparticle. This chain reaction may continue until equilibrium is reached. According to our results of roaches' behavior, the nAuNPs treated roaches had a sudden increase in their activity during 17 days after treatment, followed by a decrease in their activity for the remaining of the observation period. This might be due to the affected signal transfer in the nervous system. Similar change in behavior based on ion transfer was reported by Hoyle [31] and Luo et al [32]. This correlation of activity and the effect of the nAuNPs on the CNS of the insect are due to how the brain of the cockroach controls its muscle response and locomotion [6]. There is a significant decrease in activities after 23 days which can be attributed to changes in noise and vibration in the building. Although proteins do not break into ions, introducing charged particles into the nervous system causes an imbalance in the signal transmission that links to the insect's locomotion.

## Conclusions

We injected nAuNPs into *Blaberus Discoidalis *in order to study the interactions between particles and the roach's nervous system. *In vivo *studies showed that the nAuNPs were adapted by the roach and transferred inside the nerve cord within 17 days. After that the nAuNPs were encapsulated by the proteins present in the nervous system.

The method proposed here is an inexpensive and reliable way of observing how biological systems respond to nanoparticles in-vivo. It opens new avenues to further understand how nanoparticles affect neural communication and to treat and repair damaged nerves.

The methodology used here was proven effective to introduce nanoparticles into the nervous system and to conduct in situ characterization. There were 67% of treated roaches and 78% of untreated roaches alive after two months of treatment which indicates no major impact on the life expectancy of the cockroach for the two-month duration of this study. A longer observation period would be necessary in the future to assess the impact of nAuNPs on the average cockroach life.

## Abbreviations

CNS means the entral nervous system. The nAuNPs is for short as negatively charged gold nanoparticles. The SEG is the sub esophageal ganglion.

## Competing interests

The authors declare that they have no competing interests.

## Authors' contributions

AR designed the experiments, performed the confocal imaging, analyzed data, and drafted the manuscript. YZ extracted, fixed, and cryocut the cockroach's brains. JMG reared and collected the insects, injected the nanoparticles, and monitored food and water for the duration of the experiment. SK fabricated the nanoparticles and performed the TEM imaging. SBV and HL conceived research and approaches, participated in writing. All authors read and approved the final manuscript.
